# The correlation beteen lifestyle and risk of metabolic syndrome in schizophrenia

**DOI:** 10.1192/j.eurpsy.2023.2262

**Published:** 2023-07-19

**Authors:** K. Krysta, B. Tredzbor, E. Martyniak, K. Piekarska-Bugiel, A. Koźmin-Burzyńska, A. Cieślik, M. Krzystanek

**Affiliations:** 1 Department of Rehabilitation Psychiatry; 2 Department of Psychiatry and Psychotherapy, Medical University of Silesia, Katowice, Poland

## Abstract

**Introduction:**

Patients suffering from schizophrenia have a higher risk of premature death. An unhealthy lifestyle contributes to increased risk of cardiovascular diseases, metabolic syndromes, suicides among them. In addition to the use of selected therapy with a restriction metabolic risk has become important to influence non-pharmacological factors such as proper diet, introducing the principles of a healthy lifestyle. A diet rich in fiber, the DASH diet, the Mediterranean diet may become beneficial in terms of lowering parameters metabolic, cardiovascular and immune related to premature mortality in schizophrenia.

Patients suffering from schizophrenia have a higher risk of premature death. An unhealthy lifestyle contributes to increased risk of cardiovascular diseases, metabolic syndromes, suicides among them. In addition to the use of selected therapy with a restriction metabolic risk has become important to influence non-pharmacological factors such as proper diet, introducing the principles of a healthy lifestyle. A diet rich in fiber, the DASH diet, the Mediterranean diet may become beneficial in terms of lowering parameters metabolic, cardiovascular and immune related to premature mortality in schizophrenia.

**Objectives:**

The objective of this study was to evaluate the influence of the lifestyle on the metabolic parameters in schizophrenia

**Methods:**

In our study, we assessed the influence of diet, nutritional knowledge and lifestyle on parameters of metabolic syndrome (cholesterol, triglicerydes, glucose) in patients with schizophrenia.

**Results:**

In the results we have found positive co-relations between unhealthy diet and lifestyle and lack of knowledge on proper nutrition and increased parameters of metabolic syndrome.

**Table:** 

Groups	BMI (Std. Dev)	Cholesterol HDL mg/dl	Triglicerides mg/dl	Insuline resistanece	Insuline uU/ml
Study group	30,58(4,44)	45,63(7,34)	177,32(108,76)	4,68(4,64)	19.77(17,35)
Control Group	26,00(3,39)	54,32(14,07)	111,47(57,48)	2,1(!,21)	9,01(4,97)

**Conclusions:**

Dietary intervention may become one of the therapeutic goals in schizophrenia.

**Disclosure of Interest:**

None Declared

